# Involvement of sulfoquinovosyl diacylglycerol in DNA synthesis in *Synechocystis *sp. PCC 6803

**DOI:** 10.1186/1756-0500-5-98

**Published:** 2012-02-16

**Authors:** Motohide Aoki, Mikio Tsuzuki, Norihiro Sato

**Affiliations:** 1 School of Life Sciences, Tokyo University of Pharmacy and Life Sciences, Horinouchi 1432-1, Hachioji, Tokyo 192-0423, Japan; 2 JST, CREST, 5, Sanbancho, Chiyoda-ku, Tokyo 102-0075, Japan

## Abstract

**Background:**

Sulfoquinovosyl diacylglycerol (SQDG) is present in the membranes of cyanobacteria and their postulated progeny, plastids, in plants. A cyanobacterium, *Synechocystis *sp. PCC 6803, requires SQDG for growth: its mutant (SD1) with the *sqdB *gene for SQDG synthesis disrupted can grow with external supplementation of SQDG. However, upon removal of SQDG from the medium, its growth is retarded, with a decrease in the cellular content of SQDG throughout cell division, and finally ceases. Concomitantly with the decrease in SQDG, the maximal activity of photosynthesis at high-light intensity is repressed by 40%.

**Findings:**

We investigated effects of SQDG-defect on physiological aspects in *Synechocystis *with the use of SD1. SD1 cells defective in SQDG exhibited normal photosynthesis at low-light intensity as on culturing. Meanwhile, SD1 cells defective in SQDG were impaired in light-activated heterotrophic growth as well as in photoautotrophic growth. Flow cytometric analysis of the photoautotrophically growing cells gave similar cell size histograms for the wild type and SD1 supplemented with SQDG. However, the profile of SD1 defective in SQDG changed such that large part of the cell population was increased in size. Of particular interest was the microscopic observation that the mitotic index, i.e., population of dumbbell-like cells with a septum, increased from 14 to 29% in the SD1 culture without SQDG. Flow cytometric analysis also showed that the enlarged cells of SD1 defective in SQDG contained high levels of Chl, however, the DNA content was low.

**Conclusions:**

Our experiments strongly support the idea that photosynthesis is not the limiting factor for the growth of SD1 defective in SQDG, and that SQDG is responsible for some physiologically fundamental process common to both photoautotrophic and light-activated heterotrophic growth. Our findings suggest that the SQDG-defect allows construction of the photosynthetic machinery at an elevated level for an increase in cell mass, but represses DNA synthesis. SQDG may be essential for normal replication of chromosomal DNA for completion of the cell cycle.

## Background

The membranes of cyanobacteria and plastids in plants are similar to include three glycolipids, monogalactosyl diacylglycerol (MGDG), digalactosyl diacylglycerol (DGDG) and SQDG, and a phospholipid, phosphatidylglycerol (PG) [1]. We have revealed physiological significance of SQDG in a cyanobacterium, *Synechocystis *sp. PCC 6803, and a green alga, *Chlamydomonas reinhardtii*, through characterization of their respective mutants deficient in SQDG synthesis [2,3]. First, SQDG was found to contribute to the functional and/or structural integrity of the photosystem II complex in thylakoid membranes by associating with the complex. This observation is consistent with the positioning of SQDG in the PSII complex in a cyanobacterium, *Thermosynechococcus elongatus*, observed on X-ray analysis of the complex [4]. In contrast, another cyanobacterium, *Synechococcus *sp. PCC 7942, and a seed plant, *Arabidopsis thaliana*, showed no remarkable impairment in photosynthesis when the genes for SQDG synthesis were disrupted [3,5,6]. Distinct from its role as a membrane lipid, our second finding was that SQDG is utilized as a sulfur (S)-storage lipid during S-starvation in green algae such as *C. reinhardtii*, which degrades SQDG to ensure a major internal sulfur-source for protein synthesis [7,8]. Despite the induction of degradation of SQDG, SQDG synthesis capacity was increased in *C. reinhardtii *to maintain SQDG at 5% of the initial level throughout the period of sulfur-starvation [7]. It seemed that the minor level of SQDG, as a membrane lipid, stabilizes the structure of the PSI complex [8]. In contrast, *A. thaliana *does not positively degrade SQDG when exposed to S-deficient stress [9]. SQDG has thus been revealed to play different roles depending on the species, despite its conservation among oxygenic photosynthetic organisms [1]. Of particular interest in this context is that SQDG is required for the growth of *Synechocystis*, but not for that of either *Synechococcus*, *C. reinhardtii *or *A. thaliana *[3,5,6,10].

Indispensability of galactolipids or PG for normal growth has been reported in some photosynthetic organisms. A knockout mutant of MGDG synthase 1 isolated from *A. thaliana *showed almost complete loss of both MGDG and DGDG, disruption of photosynthetic membranes, and impairment in photoautotrophic growth of the seedling [11]. These lines of evidence led to a conclusion that these galactolipids are responsible for construction of photosynthetic apparatus. Interestingly, this mutant was impaired also in embryo development, which suggested some essential role of MGDG that must be independent of the photosynthetic dysfunction [11]. *Synechocystis *mutants with the genes for PG synthesis disrupted were lethal, and required supplementation of PG in the culture for the growth [12,13]. PG was found to contribute to the structural and functional integrity of the PSI and PSII complexes in this cyanobacterium [14,15]. Indispensability of PG in *A. thaliana *was also shown for growth, through characterization of the mutants defective in the genes for PG synthesis [16,17]. PG seemed to contribute to the development of chloroplasts and, in particular, of thylakoid membranes [16,17]. Although evidence has thus accumulated for the roles of the membrane lipids, which are essential for growth or physiological processes such as photosynthesis, no answer has been obtained as to a question of what leads directly to the lethal phenotype of these lipid mutants.

We here explored the role of SQDG that is essential for growth of *Synechocystis*, by examining the effects of the SQDG-defect on photosynthesis and respiration on culturing, and also on cell size, and the Chl and DNA contents of individual cells with use of a flow cytometer. From the results obtained, we proposed the importance of SQDG for DNA synthesis and eventually for completion of the cell cycle.

## Methods

### Culture conditions

Wild type of Synechocystis sp. PCC 6803 and its mutant, SD1, deficient in SQDG synthesis were grown photoautotrophically in BG-11 medium, as we previously reported [3]. For light-activated heterotrophic growth (LAHG), the cells were cultured in the BG-11 medium supplemented with 10 mM glucose and illumination with a pulse of low light (10 μEin/m^2^·s) for 5 min each day [18]. SQDG prepared from the wild type cells of *Synechocystis *sp. PCC 6803 was sonicated in the growth medium for liposome production (5 mM) and filtered for sterilization. The amount of SQDG that was added to the culture medium was 20 μM. Growth of the cells was monitored by measurement of the optical density at 730 nm.

### Measurement of photosynthetic and respiratory activities

Photosynthetic O_2 _evolution (net photosynthesis) or respiratory O_2 _uptake was measured at 30°C for intact cells equivalent to 2.5 μg Chl·ml^-1 ^in the culture medium containing 10 mM NaHCO_3_, with the use of a Clark-type oxygen electrode (Rank Brothers, Cambridge, UK), according to [3]. The reaction mixture for photosynthesis was illuminated with a tungsten projector lamp at the indicated light intensity, while that for respiration was kept in the dark.

### Nomarski microscopic examination

Cells were observed under a Nomarski microscope (BX50; Olympus, Tokyo, Japan) equipped with an image-capture camera (DP50; Olympus, Tokyo, Japan). ImageJ software version 1.44 provided with NIH (http://rsb.info.nih.gov/ij/) was used for size analysis of cells in the images.

*Flow cytometry analyses of cell size, chlorophyll autofluorescence and DNA content*. Flow cytometry (FACSort, instrument with LYSIS II software, Becton Dickinson, NJ, USA) was performed for determination of the cell size, chlorophyll autofluorescence intensity and DNA content. Forward scattering (FSC), side scattering (SSC), and fluorescence (FL) were sdetected by irradiation with a laser at 488 nm. FSC and SSC reflect the size and complexity of a cell, respectively. FL was detected as FL1 (530 ± 15 nm), FL2 (585 ± 21 nm), and FL3 (over 650 nm). FL3 reflects chlorophyll autofluorescence. As size standards, polystyrene beads (#F-13838; Molecular Probes, OR, USA) were used. For determination of the DNA content, collected cells were fixed with 100% MeOH, passed through a 25 gauge syringe (in order to prevent aggregation), and then washed twice with phosphate-buffered saline. The suspension of fixed cells was treated with RNase (20 μg/ml, final concentration) for more than 30 min at room temperature, and thereafter stained with 50 μg/ml (final concentration) propidium iodide (PI) [19]. FL2 was used for determination of PI-stained DNA content of the cells. Ten thousand events were acquired for each assay. Acquired data were analyzed and visualized with CellQuest software version 3.1 (Becton Dickinson, NJ, USA) or WinMDI version 2.9 (http://facs.scripps.edu/software.html, The Scripps Research Institute, CA, USA) for 2D color density plot analyses.

## Results and discussion

### Contribution of SQDG to both photoautotrophic and light-activated heterotrophic growth

**Figure 1:**
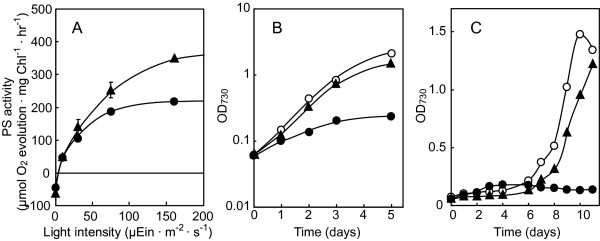
**Effect of the SQDG-defect on the light-intensity dependency of photosynthesis or LAHG in SD1**. (A) SD1 cells precultured with supplementation of SQDG were transferred to SQDG-replete or -deprived conditions for 3 days. Then, photosynthetic activity was measured with illumination with different light intensities. The values are averages ± SD of three independent measurements. Closed circles, SD1-SQDG; closed triangles, SD1 + SQDG. (B) Wild-type and SD1 cells were precultured under photoautotrophic conditions with supplementation of SQDG. Wild type cells were further grown under the same conditions, while SD1 cells were grown under photoautotrophic conditions with or without SQDG-supplementation. Open circles, wild type; closed circles, SD1-SQDG; closed triangles, SD1 + SQDG. (C) Wild-type and SD1 cells were precultured under LAHG conditions with supplementation of SQDG. Wild type cells were further grown under the same conditions, while SD1 cells were grown under LAHG conditions with or without SQDG-supplementation. Open circles, wild type; closed circles, SD1-SQDG; closed triangles, SD1 + SQDG.

The SD1 mutant of *Synechocystis*, which has the *sqdB *gene for SQDG synthesis disrupted, requires external supplementation of SQDG for its growth. However, even after a shift to SQDG-free medium, the SD1 cells that had incorporated SQDG in advance could continue to grow for four days although at a much reduced rate. Eventually, the OD_730 _value became double the initial level (Figure 1B, [3]), the relative content of SQDG decreasing by five-fold [3]. To determine whether or not the growth was interfered by some damage to photosynthesis, we investigated the effect of the SQDG-defective mutation on the light-intensity dependency of photosynthesis (Figure 1C). The maximum photosynthetic activity with the high-light intensity was 40% lower for the SD1 cells depleted of SQDG than for those grown with supplementation of SQDG, as we previously reported [3]. However, under low-light conditions (30 μEin/m^2^·s), which we adopted for the culturing, the photosynthetic activity was similar for SQDG-replete and -defective SD1 cells. Moreover, the rates of respiration in the dark were also indistinguishable (0 μEin/m^2^·s). These results implied that the SQDG-defective mutation has no deleterious effect on the functioning of the photosynthetic machinery under low-light conditions or the respiratory machinery, and that the arrest of the cell growth is therefore not due to a defect in energy production or carbon fixation.

*Synechocystis *exhibits light-activated heterotrophic growth (LAHG), i.e., the cells grow heterotrophically in a BG11 medium supplemented with 10 mM glucose, when illuminated with a light-pulse for 5 min each day. We then examined whether or not SQDG is required for LAHG (Figure 1C). Normal growth was observed for SD1 cells supplemented with SQDG, but not for ones devoid of SQDG, which demonstrated that, in *Synechocystis*, SQDG is responsible for LAHG as well as for photoautotrophic growth. It is likely that SQDG is essential for some physiologically fundamental process that is common to both photoautotrophic and light-activated heterotrophic growth.

### Effects of the SQDG-defect on morphological characteristics

**Figure 2:**
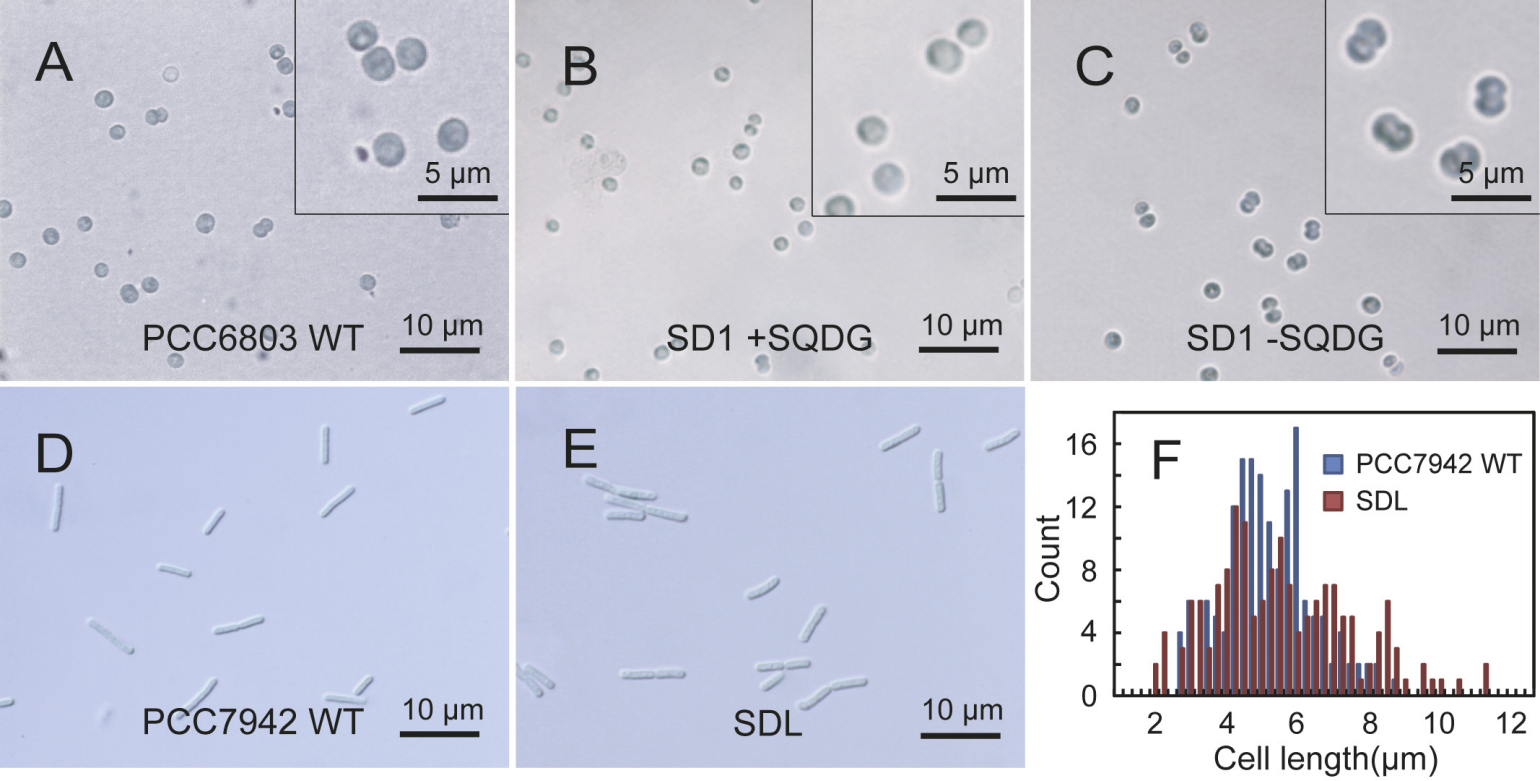
**Effect of the SQDG-defect on the cell morphology of *Synechocystis *or *Synechococcus***. Wild-type cells were normally cultured whereas SD1 cells precultured with supplementation of SQDG were transferred to SQDG-replete or -deprived conditions for 3 days. (A) Wild type cells of *Synechocystis*. (B) SD1 cells replete of SQDG. (C) SD1 cells defective in SQDG. (D) Wild type cells of *Synechococcus*. (E) SDL1 cells. Scale bars indicate 10 μm. Insets show two-fold magnified images. Shown are the results representative of three independent experiments. (F) Distribution of cell lengths in the wild type *Synechococcus *or SDL1.

**Figure 3:**
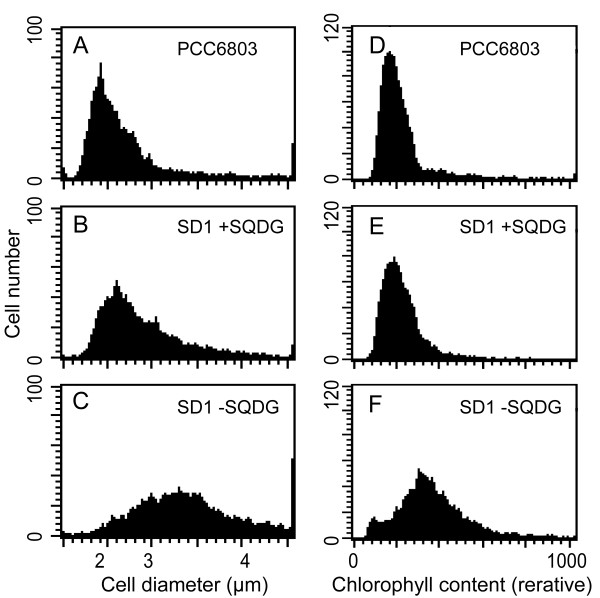
**Effect of the SQDG-defect on the cell size or Chl content**. Wild-type cells were normally cultured whereas SD1 cells precultured with supplementation of SQDG were transferred to SQDG-replete or -deprived conditions for 3 days. In individual cells of the wild type or SD1, forward light scattering or Chl fluorescence was measured with a flow cytometer, as described under Materials and Methods. Shown are the results representative of three independent experiments. (A) Wild-type cells. (B) SD1 cells replete of SQDG. (C) SD1 cells defective in SQDG.

We then examined the impact of disruption of the *sqdB *gene on cell morphology by microscopy. No significant difference was found in cell size between the wild type and SD1 supplemented with SQDG (Figure 2A, B). However, SD1 cells deprived of SQDG tended to become larger in size, quite often separated by septa, as if the cell cycle was arrested specifically at some particular stage of cell division (Figure 2C). Accordantly, the mitotic index, i.e., proportion of dumbbell-like cells with a septum, of SD1 cells defective in SQDG was 29%, and thus was quite higher than that of SD1 cells replete of SQDG (14%) or the wild type (12%). We then investigated forward light scattering for the cells with a flow cytometer: the histogram remained almost unchanged for the wild type throughout the culture for three days, there being a major cell population peak at a position corresponding to a cell diameter of about 2 μm (Figure 3A). A similar trend was observed for SD1 cells cultured in the presence of SQDG (Figure 3B). However, SD1 cells, when shifted to SQDG-free medium, showed elevated light scattering such that the average diameter of the cells were estimated to become approximately 1.7-fold higher in 3 days (Figure 3C). It thus turned out that the increased population of enlarged cells with septa led to elevation of the light scattering in an SD1 culture without SQDG. This evidence implied that SQDG, directly or indirectly, is crucial for normal completion of cell division.

The *sqdB *disruptant of *Synechococcus*, in contrast to the *Synechocystis *counterpart, previously exhibited no deleterious effect on growth [3]. To determine whether or not SQDG is responsible for the cell size of *Synechococcus*, its *sqdB *mutant (SDL1) was subjected to microscopic analysis. No obvious effect was found on the cell morphology (Figure 2D,E), cell length (5.5 ± 2.0 μm for SDL1, cf. 5.2 ± 1.3 μm for the wild type), or histogram of the cell size (Figure 2F), indicating no role of SQDG in cell division in *Synechococcus*.

### Effect of the SQDG-defect on the content of chlorophyll

A synchronized culture of *Synechococcus *sp. PCC 6301 previously exhibited the sequential appearance of periods of synthesis of macromolecules in the cell cycle, the first appearance of protein synthesis, followed by RNA, phospholipid, and DNA synthesis in that order. This highly-ordered time schedule of macromolecule synthesis suggests a coordinated genetic regulatory system for the cell cycle of *Synechococcus *sp. PCC 6301 [20,21].

To determine whether or not the cessation of cell growth of SD1 is due to impaired synthesis of macromolecules, we here investigated the cellular contents of Chl in individual cells by flow cytometry. The wild type and SD1 grown with external SQDG exhibited a similar cell population as to Chl fluorescence throughout the culturing period (Figure 3D, E). In SD1 cells depleted of SQDG, the peak shifted to a position corresponding to higher Chl fluorescence (Figure 3F), which led to a 1.4-fold increase in the average content of Chl on a cell basis. These results indicated that depletion of SQDG allowed the cells to increase the content of Chl and, therefore, those of the PSI and PSII complexes, which should be associated with Chl.

**Figure 4:**
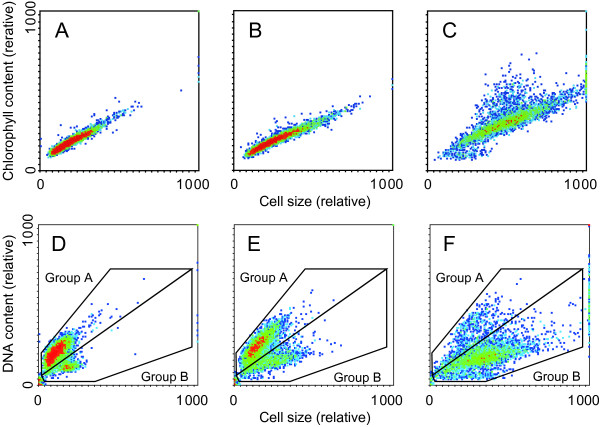
**Effect of the SQDG-defect on the cytogram of Chl fluorescence vs. cell size or DNA content vs. cell size**. Wild-type cells were normally cultured whereas SD1 cells precultured with supplementation of SQDG were transferred to SQDG-replete or -deprived conditions for 3 days. In individual cells of the wild type or SD1, Chl fluorescence vs. forward light scattering (A-C), or DNA content vs. forward light scattering (D-F) was measured with a flow cytometer, as described in Materials and methods. The results were visualized in 2D color density plot such that blue-to-red color changes in the plot indicate dot-density changes from low to high. Shown are the results representative of three independent experiments. (A), (D): Wild-type cells. (B), (E): SD1 cells replete of SQDG. (C), (F): SD1 cells defective in SQDG.

The flow-cytometric cytograms of Chl fluorescence versus cell size were similar for the wild type and SQDG-replete SD1 cells: the Chl fluorescence was almost directly proportional to the cell size, a rod-shaped cell population being formed, with a zone of high cell-density at the lower-left to central part (Figure 4A, B). However, cells of SD1 depleted of SQDG showed extreme extension of the rod-shape in the upper-right direction such that the population of the cells of large size with an elevated content of Chl was increased (Figure 4C). Since SQDG-depleted SD1 cells exibited normal specific activity of the photosynthetic machinery on the culturing (Figure 1A), the SD1 cells of large size should show high photosynthesis activity on a cell basis. Thus, the SQDG-defect did not interfere with construction of the photosynthetic machinery, which would allow the SD1 cells to synthesize macromolecules that were essential, at least, for an increase in size.

### Effect of the SQDG-defect on the content of DNA

Figure 4D shows a flow-cytometric cytogram of DNA content versus cell size. Interestingly, the wild type cells could be divided into two populations, i.e., a large population, in which the DNA content is almost directly proportional to the cell size (Group A, 86% of the cells), and a small one, consisting of cells that maintained DNA at a low level, irrespective of the cell size (Group B, 14%). Large part of the cells of *Synechocystis *(Group A) thus seemed to synthesize DNA during the whole cell cycle, as in the case of vegetative cells of *Anabaena *sp. PCC 7120 [22]. The cytogram of SD1 cells grown with SQDG (Figure 4E) was basically the same as that of the wild type: the cell population of Group A (69%) was much larger than that of Group B (31%). The larger population of Group B for SD1 than for the wild type may be due to incomplete chemical complementation by SQDG. Strikingly, an SD1 culture, when depleted of SQDG, showed a reverse in the distribution patterns of the cells such that the cell population of Group A was much reduced to 14% with pronounced elevation of that of Group B to 86% (Figure 4F). Therefore, the SQDG-defect led to an increase in the population of cells that contained a relatively low level of DNA, but were enlarged. These results implied that the SQDG-defect represses DNA synthesis, but allows the cells to increase in mass. The probable arrest of the cell cycle at the mitotic stage for SD1 cells devoid of SQDG (Figure 2C, 3C) might be due to the inability of the cells to synthesize DNA to a proper level for completion of cell division.

### Possible mechanism for involvement of acidic lipids in DNA synthesis and cell division in Synechocystis

We here reported two novel discoveries concerning the roles of SQDG. One is the responsibility of SQDG for DNA synthesis. In bacteria, initiation of chromosomal DNA replication depends on the DnaA protein, which binds to *oriC *and multimerizes in an ATP-bound form, thereby opening up the duplex DNA so that other proteins can enter [23]. In *Escherichia coli*, acidic phospholipids in lipid bilayers promote the exchange of ADP for ATP in DnaA for generation of replicatively active ATP-DnaA [24,25]. One possible explanation for the growth arrest of SD1 cells may be the responsibility of SQDG, an acidic glycolipid, in place of acidic phospholipids, for replication of chromosomal DNA in *Synechocystis*.

In contrast, acidic phospholipids also have an inhibitory effect on DnaA-binding, which was proposed to prevent re-initiation of DNA replication until the proper time, and cardiolipin or synthetic PG with unsaturated fatty acids is effective [25]. SD1 cells, with a decrease in the SQDG content, showed an increase in the PG content up to two-fold the initial level [3]. The quantitatively reverse alteration in SQDG and PG maintains the total amount of acidic lipids at a certain level in cyanobacterial membranes, thus is regarded as reflecting the requirement of a balanced negative charge in membranes for some physiological processes [1]. However, it should be noted that PG in SD1, like that in the wild type, contained 18:2 representing 18-19% of the constituent fatty acids (data not shown), indicating that as much as ca. 40% of PG molecules possess 18:2 in view of the exclusive distribution of C18 acids to the *sn*-1 position in *Synechocystis *[1]. The accumulation of excess highly unsaturated PG in SD1 cells might resultantly perturb the initiation of chromosomal DNA replication by DnaA, thereby retarding DNA synthesis. The regulatory mechanism for DNA synthesis seems to have evolved to involve SQDG in *Synehocystis*, no matter whether its action is direct or indirect.

The other novel discovery is the responsibility of SQDG for the progression of cell division, which we judged from the increase in cell size as well as in the mitotic index, in SD1 cells defective in SQDG. Evidence has accumulated for bacteria that membrane lipids participate in cell division by interacting with proteins in the cell division machinery such as MinD [26]. Apart from its possible contribution to DNA synthesis, SQDG or PG might participate directly in some machinery component of cell division so that a defect in SQDG or a concomitant increase in the PG content perturbs the functioning of the machinery to prevent completion of cell division. In any case, concerning their roles in DNA synthesis or cell division, SQDG and PG can never substitute for each other in *Synechocystis*. A future work will be to specify whether it is a defect in SQDG or a concomitant increase in PG that impairs DNA synthesis and cell division. One possible strategy to answer this question may be to investigate how the DNA content and cell cycle progression are affected in SD1 when the unsaturation level of PG is lowered by disruption of the desaturase genes [27].

The membrane lipids of cyanobacteria, similar to plastids, are composed mainly of three glycolipids and PG [1], thus being quite distinct from the membrane lipids in non-photosynthetic prokaryotic and eukaryotic organisms such as *E. coli*, in which phospholipids predominate. The characteristic lipid composition of the oxygenic photosynthetic organisms seems reasonable in view of their physiological and ecological aspects, i.e. their ability to ensure sugar by photosynthesis and limited availability of phosphorus-source in their environment. The regulatory mechanism for the cell cycle might have evolved to show quite a novel dependency on SQDG in *Synechocystis*, in accordance with its unique lipid composition.

Since *Gloeobacter violaceus *PCC 7421, which is regarded as the most primitive type of cyanobacterium, does not contain SQDG, cyanobacteria seem to have acquired an SQDG synthetic system through evolution within cyanobacteria [1]. Intriguingly, the roles of SQDG in *Synechocystis *postulated above do not hold true for *Synechococcus*, and thus at least two mechanisms for progression of the cell cycle might have evolved in cyanobacteria, with respect to the dependency on SQDG. Our novel discovery of involvement of SQDG in progression of the cell cycle in *Synechocystis *will greatly facilitate understanding of how the regulatory mechanism for the cell cycle has developed, especially, how SQDG has been integrated into it, through evolution within cyanobacteria, and how the cell-cycle dependency on SQDG has been attained through the evolution of cyanobacteria into plastids in plants. Moreover, our study will provide an opportunity for researchers to examine whether or not membrane lipids other than SQDG participate in cell cycle progression of cyanobacteria.

## Conclusions

We have found strong supportive evidence for responsibility of SQDG in *Synechocystis *for physiologically fundamental process that is not dependent on photosynthesis, but is common to both photoautotrophic and light-activated heterotrophic growth. On the basis of our flow-cytometric analysis, it is tempting to speculate that SQDG is involved in DNA replication, and eventually, in cell cycle progression. However, it will be necessary to unravel the elementary process during DNA replication, in which SQDG participates, and the corresponding protein(s) SQDG interact with, in order to specify the role of SQDG at the molecular level.

## Competing interests

The authors declare that they have no competing interests.

## Authors' contributions

MA measured photosynthesis and respiration, and carried out microscopic and flow-cytometric analysis. MA, MT, and NS conceived of the study, participated in its design, and drafted the manuscript. All authors read and approved the final manuscript.
